# Clinical evaluation of CEA, CA19-9, CA72-4 and CA125 in gastric cancer patients with neoadjuvant chemotherapy

**DOI:** 10.1186/1477-7819-12-397

**Published:** 2014-12-29

**Authors:** Zhipeng Sun, Nengwei Zhang

**Affiliations:** Oncology Surgery Department, Capital Medical Cancer Centre, Beijing Shijitan Hospital, Capital Medical University, Beijing, 100038 China; Beijing Shijitan Hospital, Capital Medical University, Room 334, Administrative Building, Beijing, 100038 China

**Keywords:** Gastric cancer, Neoadjuvant chemotherapy, CEA, CA19-9, CA72-4, CA125

## Abstract

**Background:**

In the clinical practice of neoadjuvant chemotherapy, response markers are very important. We aimed o investigate whether tumor markers CEA(carcino-embryonic antigen), CA19-9(carbohydrate antigen 19–9), CA72-4(carbohydrate antigen 72–4), and CA125(carbohydrate antigen 125) can be used to evaluate the response to neoadjuvant chemotherapy, and to evaluate the diagnosis and prognosis value of four tumor markers in the patients of gastric cancer.

**Methods:**

A retrospective review was performed of 184 gastric cancer patients who underwent a 5-Fu, leucovorin, and oxaliplatin (FOLFOX) neoadjuvant chemotherapy regimen, followed by surgical treatment. Blood samples for CEA, CA19-9, CA72-4, and CA125 levels were taken from patients upon admission to the hospital and after neoadjuvant chemotherapy. Statistical analysis was performed to identify the clinical value of these tumor markers in predicting the survival and the response to neoadjuvant chemotherapy.

**Results:**

Median overall survival times of pretreatment CA19-9-positive and CA72-4-positive patients (14.0 +/−2.8 months and 14.8 +/−4.0 months, respectively) were significantly less than negative patients (32.5 +/−8.9 months and 34.0 +/−10.1 months, respectively) (*P* = 0.000 and *P* = 0.002, respectively). Pretreatment status of CA19-9 and CA72-4 were independent prognostic factors in gastric cancer patients (*P* = 0.029 and *P* = 0.008, respectively). Pretreatment CEA >50 ng/ml had a positive prediction value for clinical disease progression after neoadjuvant chemotherapy according to the ROC curve (AUC: 0.694, 95% CI: 0.517 to 0.871, *P* = 0.017). The decrease of tumor markers CEA, CA72-4, and CA125 was significant after neoadjuvant chemotherapy (*P* = 0.030, *P* = 0.010, and *P* = 0.009, respectively), especially in patients with disease control (including complete, partial clinical response, and stable disease) (*P* = 0.012, *P* = 0.020, and *P* = 0.025, respectively). A decrease in CA72-4 by more than 70% had a positive prediction value for pathologic response to neoadjuvant chemotherapy according to the ROC curve (AUC: 0.764, 95% CI: 0.584 to 0.945, *P* = 0.020).

**Conclusions:**

Our results suggest that high preoperative serum levels of CA72-4 and CA19-9 are associated with higher risk of death, high pretreatment CEA levels (>50 ng/ml) may predict clinical disease progression after neoadjuvant chemotherapy, and a decrease (>70%) of CA72-4 may predict pathologic response to neoadjuvant chemotherapy.

## Background

Gastric cancer is the fourth most common cancer worldwide and the second most frequent cause of cancer death, affecting about one million people per year [1]; surgery remains the only cure for this disease. However, in a recent study, the disease was too far advanced to receive curative resection in more than 30% of surgical patients [2]. Despite efforts for early detection, most patients with gastric cancer in Europe and the United States continue to present with advanced stages of the disease, therefore the current efforts to improve survival in patients with advanced gastric carcinoma aim to increase the rate of complete tumor removal. Preoperative neoadjuvant chemotherapy is an available option to achieve these goals [3].

Neoadjuvant chemotherapy is cancer treatment in which a drug is given to the patient prior to cancer surgery or radical radiotherapy, and it is mostly used in cases where surgery is planned in the future. The surgical interest in neoadjuvant therapy for gastric cancer is driven by a long-standing frustration with the poor outcomes achieved with surgery alone, as well as when surgery is followed by intense adjuvant chemotherapy and radiotherapy [4]. There are several studies which indicate that neoadjuvant chemotherapy could increase the rate of complete tumor resections, combat systemic metastasis, and prolong survival with tolerable and manageable toxicity, without increasing the mortality or morbidity of surgery in gastric cancer patients [3, 5, 6].

If particular patients not benefiting from preoperative treatment, who are suggested to have a prognosis inferior to surgery alone, could be identified, alternative therapies may offered at an early stage [7]. Response markers are needed to monitor treatment, and to improve quality of life of non-responders, reduce time until surgery in non-responders, and reduce costs [8].

Recently, tumor markers CEA, CA19-9, CA72-4, and CA125 are widely used in gastric cancer patients, although reports have shown the value of tumor markers as prognostic factors, clinical studies evaluating the roles of tumor markers in monitoring of chemotherapeutic efficacy are limited [9], especially in neoadjuvant chemotherapy. In this study, we analyzed the relationship between tumor markers CEA, CA19-9, CA72-4, and CA125 and the response to neoadjuvant chemotherapy in patients with gastric cancer. The aim of the study was to determine the clinical value of tumor markers in monitoring the response to neoadjuvant chemotherapy and in predicting the prognosis of gastric cancer patients.

## Methods

The research was funded by Beijing Municipal Administration of Hospitals Clinical Medicne Development of Special Funding Support,code:XMLX201309,which was approved by the ethics committee on human research of the Beijing Shijitan Hospital in accordance with the Declaration of Helsinki.

### Patients

The medical records of 184 patients who underwent neoadjuvant chemotherapy between 2009 and 2013 at Beijing Shijitan hospital, Capital Medical University, were retrospectively reviewed. All patients had histologically confirmed gastric cancer and received surgical treatment after neoadjuvant chemotherapy; patients were excluded from the study if they demonstrated other organs insufficiencies, if they had experienced an acute event within the last threemonths (cerebral, coronary, and so forth), acute infection, or major trauma. The clinicopathologic features of these patients including age, sex, and differentiation, tumor location, depth of wall invasion, lymph node metastasis, and vascular invasion, pTNM(Pathologic Tumor-Node-Metastasis) stage and responses to neoadjuvant chemotherapy were reviewed. pTNM stages were according to histological examination of a post-operation, and were assigned in accordance with the criteria established by American Joint Committee on Cancer in 2010 [10–12]. The mean follow-up time (+/− SD) for the entire patient population was 31.1 +/−23.2 months (median: 24.6 months).

### Neoadjuvant chemotherapy

The decision to treat patients with neoadjuvant chemotherapy and the neoadjuvant regimen administered was determined by multidisciplinary tumor board discussions, tumor histology and stage, patient ability to tolerate therapy, and in accordance with National Comprehensive Cancer Network (NCCN) guidelines.

All 184 patients received neoadjuvant chemotherapy; 5-Fu, and Leucovorin, and Oxaliplatin (FOLFOX) regimen. The median number of cycles administered was three (range: one to ninecycles). In all cases, surgery was performed after neoadjuvant chemotherapy.

### Evaluation of clinical and pathologic response

In this study, both clinical and pathologic responses are used to evaluate the response to neoadjuvant chemotherapy. Clinical responses to neoadjuvant chemotherapy were determined by RECIST 1.1 response evaluation criteria in solid tumors version 1.1 [13], and according to radiological examination, including ultrasonography, computed tomographic scan, upper gastrointestinal endoscopic assessment, and upper gastrointestinal tract radiography.

Pathologic responses to neoadjuvant chemotherapy were determined by the criteria defined by the MD Anderson center [14–16], and according to the histological examination of post-operation, board-certified pathologists who specialize in gastrointestinal malignancies reviewed all specimens.

### Serum assays for CEA, CA19-9, CA72-4, and CA125

Serum samples for CEA, CA19-9, CA72-4, and CA125 levels were measured at baseline and after neoadjuvant chemotherapy respectively. Serum levels of CEA, CA19-9, and CA72-4 were assayed with electrochemiluminescence (ECL) method (E170, Roche Diagnostics, Rotkreuz,Switzerland), and CA125 was measured with enzyme-linked immunosorbent assay (ELISA, CA125-ELISA-Kit, CanAg, Gothenburg,Sweden).

The cut-off levels were 5.0 ng/ml, 37.0 U/ml, 6.7 U/ml, and 20.0 U/ml for CEA, CA19-9, CA72-4, and CA125, as recommended by the manufacturer. A result was considered positive when the marker serum level was higher than the cut-off value. Positive combined detection for four tumor markers was defined as one or more tumor markers above the cut-off levels.

### Statistical analysis

Analyses were performed using SPSS 16.0 for Windows (SPSS Inc., Chicago,United States). Chi-square analysis was applied to determine the association between tumor markers and clinicopathologic features. Survival rates were defined in univariate analysis according to the Kaplan-Meier method, and log-rank test was used to assess statistical differences. The independent prognostic values of each tumor marker and clinicopathologic aspects which significantly affect survival were assessed by Cox multivariate analysis. T-test was applied to determine the differences between mean levels of tumor markers before and after neoadjuvant chemotherapy. Correlations were assessed using the Spearman rank order correlations. Receiver-operating characteristics (ROC) curve was used to evaluate the ability of tumor markers to predict the response to neoadjuvant chemotherapy. Differences were considered statistically significant when the *P* value was <0.05.

## Results

### The diagnosis and prognosis value of pretreatment levels of four tumor markers

The pretreatment levels of CEA, CA19-9, CA72-4, and CA125 were above the cut-off levels in 37.9, 28.7, 36.4, and 26.4% of cases, respectively. A total of 108 patients showed positivity for one or more tumor markers (overall sensitivity: 63.9%).

Table 1 shows the correlation between pretreatment status of tumor markers and different clinicopathological parameters. CEA was more frequently positive in the patients with lymph node involvement, well differentiation, and cardiac carcinoma (*P* = 0.005, *P* = 0.001, and *P* = 0.049, respectively), CA72-4 was more frequently positive in the patients with advanced tumor stage and vascular invasion (*P* = 0.007 and *P* = 0.006, respectively), and positive levels of combined detection were related to deeper tumor invasion, lymph node metastasis, and advanced tumor stage (*P* = 0.041, *P* = 0.014, and *P* = 0.040, respectively).

**Table 1:** **Association of pre-therapy status of tumor markers with clinicopathological parameters**

Variable	Cases (N)	CEA (+) N (%)	***P***value	CA199 (+) N (%)	***P***value	CA72-4 (+) N (%)	***P***value	CA125 (+) N (%)	***P***value	Combined detection (+) N (%)	***P***value
Gender:											
Male	127	47(39.8)		32(28.1)		37(35.6)		14(23.3)		78(66.1)	
Female	57	17(33.3)	0.424	15(30.0)	0.801	18(38.3)	0.748	9(33.3)	0.328	30(58.8)	0.366
Age(years):											
≤60	92	29(33.3)		25(29.1)		31(40.8)		9(22.0)		52(59.8)	
>60	92	35(42.7)	0.210	22(28.2)	0.903	24(32.0)	0.262	14(30.4)	0.370	56(68.3)	0.249
Differentiation:											
Moderately	30	18(62.1)		10(38.5)		5(20.0)		4(28.6)		21(72.4)	
Poorly	75	20(27.4)	0.001	15(20.8)	0.077	24(36.9)	0.124	8(22.2)	0.918	39(53.4)	0.079
Other	29										
Missing value:	50										
Tumorlocation											
Cardiac carcinoma	47	22(47.8)		11(24.4)		11(29.7)		1(6.2)		32(69.6)	
Other locations	114	34(31.2)	0.049	28(26.7)	0.776	39(38.6)	0.336	15(24.6)	0.207	64(58.7)	0.204
Missing value	23										
Invasion depth(pathological):											
T1 + T2	29	8(30.8)		5(20.8)		5(21.7)		4(28.6)		12(46.2)	
T3 + T4	146	53(39.6)	0.399	39(29.8)	0.372	47(39.5)	0.106	17(25.0)	1.000	90(67.2)	0.041
Missing value	9										
Lymph node metastasis (pathological):											
Negative	44	8(20.0)		7(18.4)		9(25.7)		5(26.3)		19(47.5)	
Positive	131	54(45.0)	0.005	37(31.6)	0.117	42(39.3)	0.147	16(25.4)	1.000	83(69.2)	0.014
Missing value	9										
Vascular invasion (pathological)											
Negative	101	32(35.6)		24(27.6)		21(26.2)		14(29.8)		54(60.0)	
Positive	66	26(41.9)	0.426	19(30.6)	0.685	27(49.1)	0.006	7(21.9)	0.435	43(69.4)	0.238
Missing value	17										
pTNM stage:											
I + II	53	13(27.1)		10(21.3)		9(20.5)		7(26.9)		25(52.1)	
III + IV	126	50(43.1)	0.055	36(32.1)	0.168	45(44.1)	0.007	14(24.1)	0.785	80(69.0)	0.040
Missing value	5										
Clinical response:											
PR + SD	137	48(36.9)		35(27.6)		40(34.5)		16(24.6)		82(63.1)	
PD	14	10(71.4)	0.012	4(28.6)	1.000	4(30.8)	1.000	3(50.0)	0.389	11(78.6)	0.391
Missing value	33										
Pathologicresponse:											
Complete and partial response	84	7(24.1)		6(21.4)		9(32.1)		4(25.0)		14(48.3)	
Noresponse	33	31(39.2)	0.145	21(26.6)	0.590	24(32.9)	0.944	12(26.1)	1.000	52(65.8)	0.097
Missing value	67										

Positive of combined detection for four tumor markers was defined as one or more tumor markers were above the cut-off levels.

**Figure 1:**
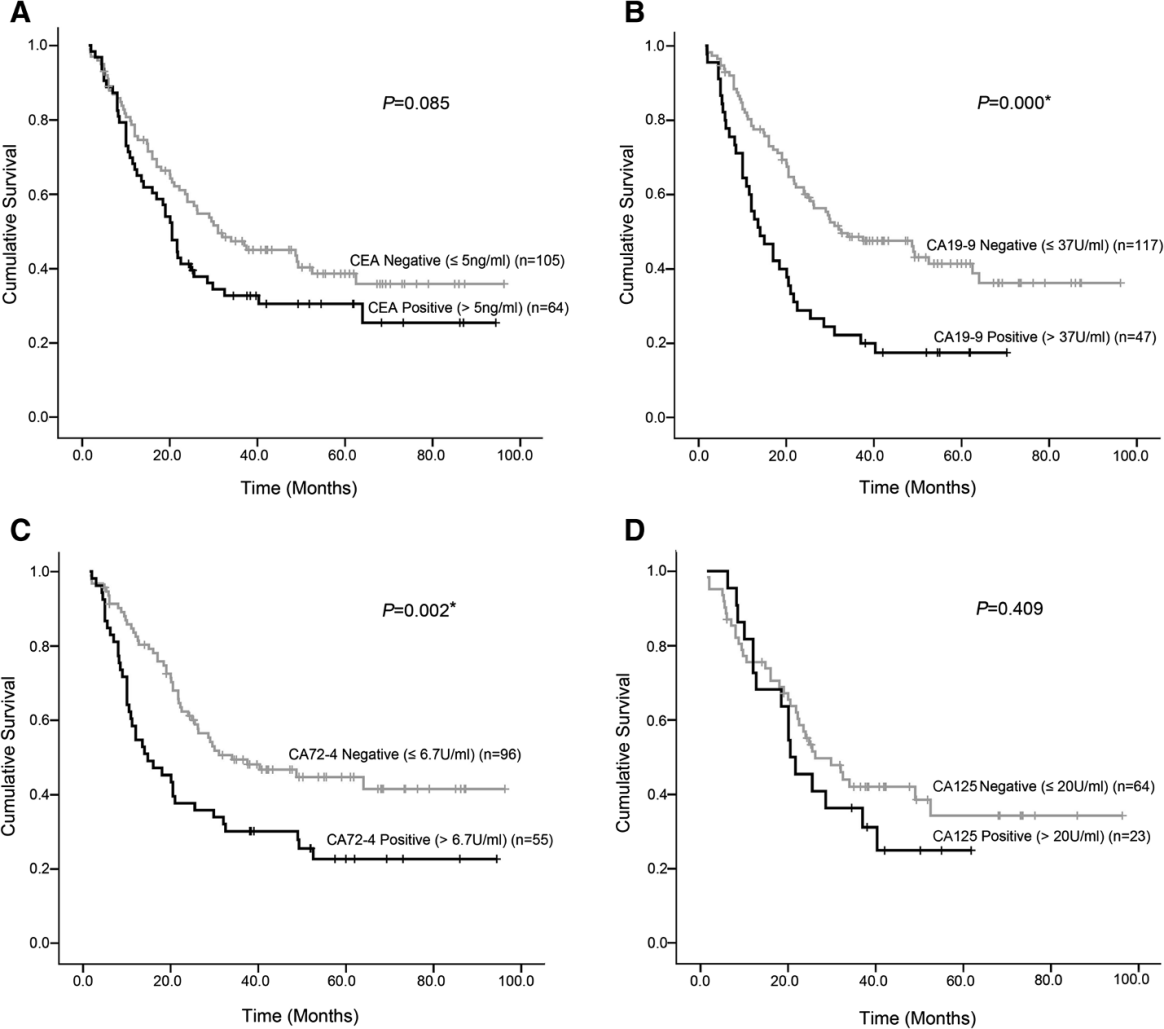
**Survival curves of patients according to CEA, CA199, CA724, and CA242 pretreatment serum positivity. A**. The difference between CEA-negative patients and CEA-positive patients was not statistically significant (*P =* 0.085). **B**. The difference between CA19-9-negative patients and CA19-9-positive patients was statistically significant (*P* = 0.000). **C**. The difference between CA72-4-negative patients and CA72-4-positive patients was statistically significant (*P* = 0.002). **D**. The difference between CA242-negative patients and CA242-positive patients wasnot statistically significant (*P* = 0.409).

Survival curves of patients according to pretreatment levels of four tumor markers are shown in Figure 1. Significant differences in survival rates were observed for CA19-9, CA72-4 (log-rank test: *P* = 0.000 and *P* = 0.002, respectively), a worse prognosis was observed in positive cases. According to the survival curve for CEA and CA125, positive cases also showed poor prognosis, but the difference did not reached statistical significance (*P* = 0.085 and *P* = 0.409, respectively).

Furthermore, to evaluate the potential of four tumor markers as independent predictors for overall survival of gastric cancer, univariate and multivariate analyses were performed (Table 2). Univariate Cox regression analysis was used to identify the factors which were significantly associated with overall survival. Selected these factors into multivariate Cox regression analysis, CA19-9, CA72-4, and pTNM stage were found as independent predictors (*P* = 0.029, *P* = 0.008, and *P* = 0.001, respectively). An increased pretreatment level of CA19-9 was associated with a 2.183-fold (95% CI: 1.085 to 4.392) higher risk of death (*P* = 0.029) and an increased pretreatment level of CA72-4 was associated with a 2.500-fold (95% CI: 1.269 to 4.926) higher risk of death (*P* = 0.008).

**Table 2:** **Univariate and multivariate analyses of factors associated with survival in gastric cancer patients with neoadjuvant chemotherapy**

Variable	Univariate analysis		Multivariate analysis	
	Hazard ratio (95% CI)	***P***value	Hazard ratio (95% CI)	***P***value
CA19-9				
Positive versusnegative	2.198 (1.459-3.311)	0.000	2.183 (1.085-4.392)	0.029
CA72-4				
Positive versusnegative	1.887 (1.241-2.869)	0.003	2.500 (1.269-4.926)	0.008
Vascular invasion				
Positive versus negative	2.401 (1.616-3.567)	0.000	1.564 (0.817-2.995)	0.177
TNM (pathological)				
III + IV versusI + II	6.041 (3.295-11.074)	0.000	5.066 (1.887-13.599)	0.001
Recurrence				
Positive versusnegative	1.745 (1.161-2.625)	0.007	0.755 (0.337-1.693)	0.495
Clinical response to NCT				
PD versus DC	2.474 (1.274-4.802)	0.007	1.103 (0.404-3.013)	0.849
Pathologic response to NCT				
pCR + pPR versus pNR	0.419 (0.218-0.805)	0.009	0.739 (0.322-1.696)	0.475

### Changes of tumor markers and correlation with response to neoadjuvant chemotherapy

All 184 patients were treated with neoadjuvant chemotherapy and clinical response to neoadjuvant chemotherapy was noted in 151 patients; complete and partial clinical response (CR and PR) was achieved in 65 (43.0%), stable disease (SD) in 72 (47.7%), and progression disease (PD) in 14 (9.3%) of patients. Pathologic response to neoadjuvant chemotherapy was noted in 117 patients; complete response (pCR) was achieved in 12 (10.3%), partial response (pPR) in 21 (17.9%), and nonresponse (pNR) in 84 (71.8%) of patients. The major toxic effects were nausea, anorexia, fatigue, anemia, thrombocytopenia, and sensory neuropathy, the major gradethree or four adverse events include anemia, and there were no treatment-related deaths.

**Figure 2:**
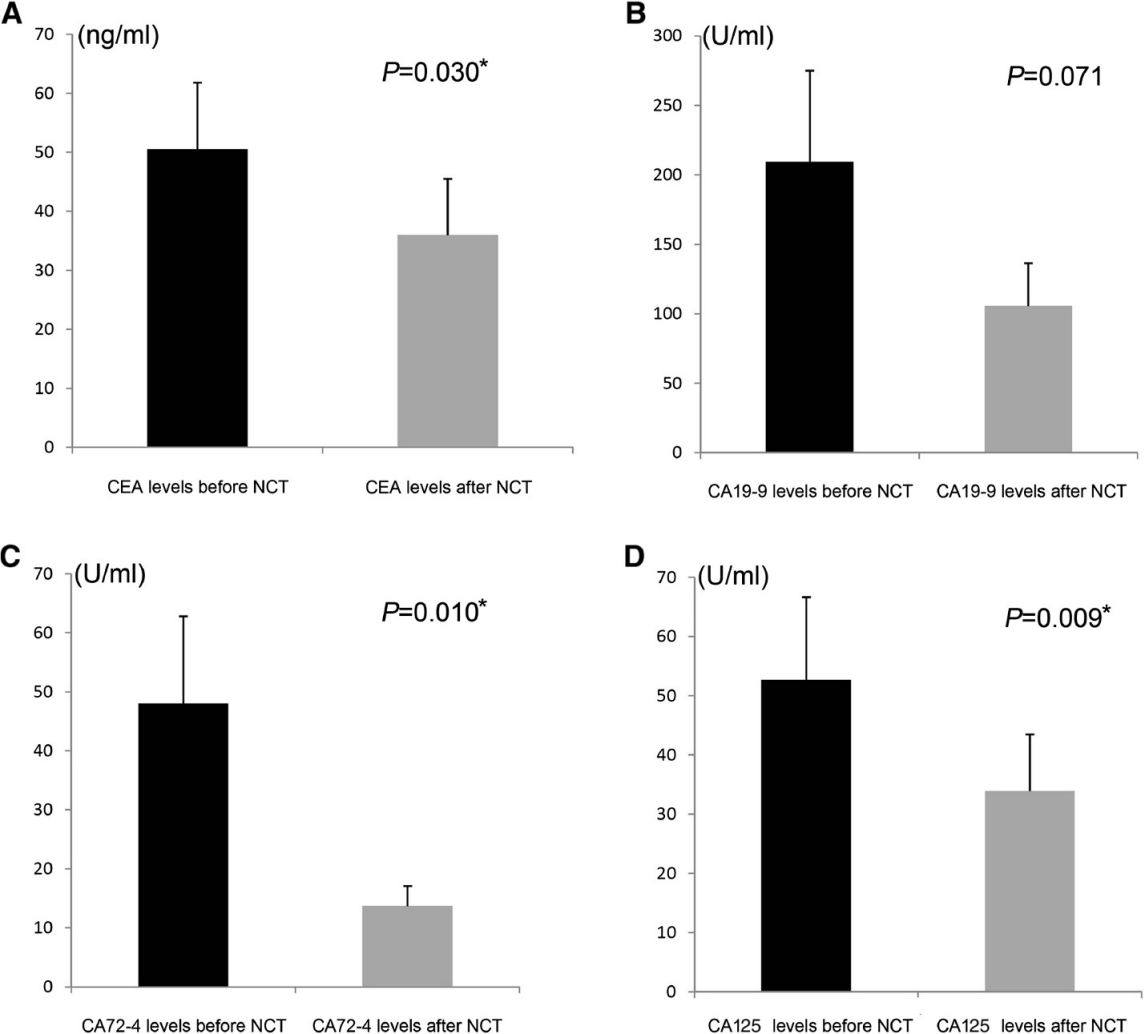
**Mean levels of four tumor markers before and after neoadjuvant chemotherapy. A**. Mean level of CEA was decreased significantly after neoadjuvant chemotherapy (*P* = 0.030). **B**. Mean level of CA19-9 was decreased after neoadjuvant chemotherapy, the difference was not statistically significant (*P* = 0.251). **C**. Mean level of CA72-4 was decreased significantly after neoadjuvant chemotherapy (*P* = 0.010). **D**. Mean level of CA242 was decreased significantly after neoadjuvant chemotherapy (*P* = 0.009).

**Figure 3:**
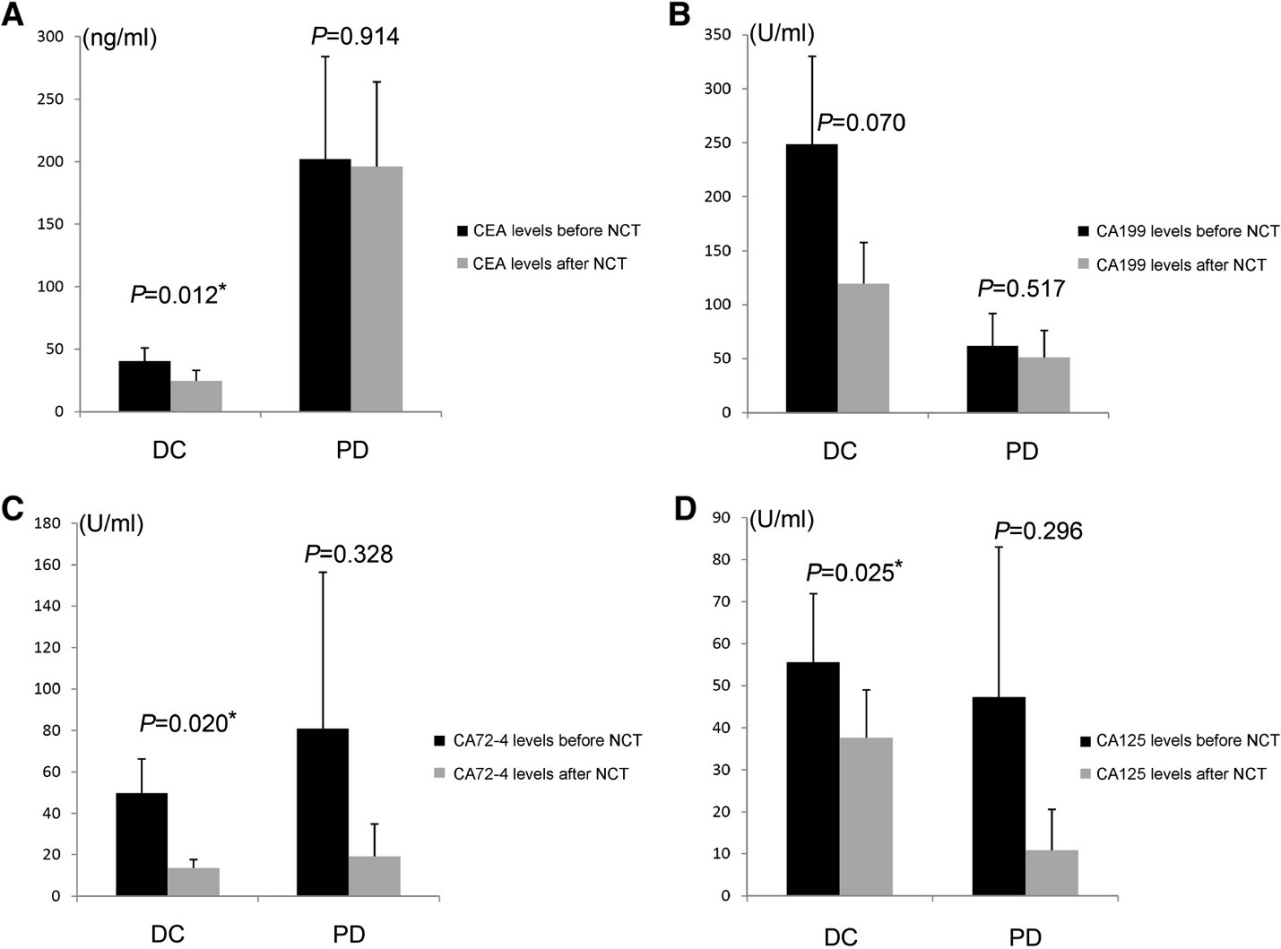
**Mean levels of four tumor markers before and after neoadjuvant chemotherapy in disease control (CR + PR + SD) and PD (disease progression) group respectively.** **A. C.** **D** shows CEA, CA724, and CA125 decreased more significantly in the disease control (CR + PR + SD) group than the disease progression (PD) group (*P* = 0.012, *P* = 0.020, and *P* = 0.025, respectively). **B.** show the decreased levels of CA199 was not statistically significant (*P* values was 0.849).

The levels of four tumor markers were recorded at baseline and after neoadjuvant chemotherapy, respectively. The mean levels of four tumor markers decreased after neoadjuvant chemotherapy, in particular, CEA, CA72-4, and CA125 decreased significantly (*P* = 0.030, *P* = 0.010, and *P* = 0.009, respectively) (Figure 2), and these three tumor markers decreased more significantly in the disease control (CR + PR + SD) group than the disease progression (PD) group (*P* = 0.012, *P* = 0.020, and *P* = 0.025, respectively) (Figure 3).

The changes of tumor markers were observed in this study, and patients were excluded if baseline serum concentration was below the upper limit of normal (ULM).

**Figure 4:**
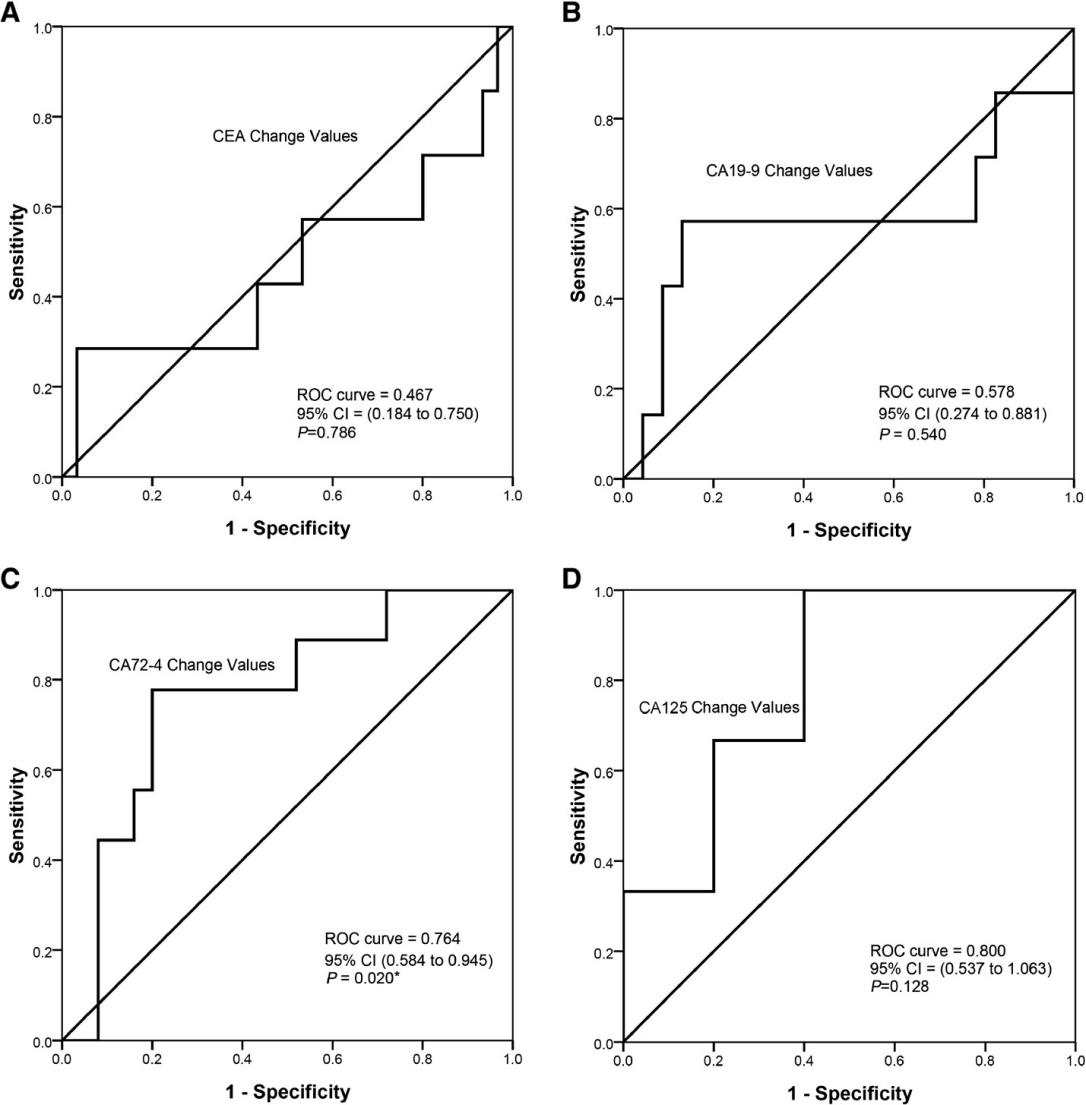
**ROC curves for the decrease of the four tumor markers to predict the disease control after neoadjuvant chemotherapy. A**, **B**. shows the decrease of CEA, CA199 was not statistically significant (*P* values were 0.785, and 0.540, respectively). **C**. The area under the ROC curve of CA72-4 change values was 0.764 (95% CI: 0.584 to 0.945, *P* = 0.020); the optimal cutoff which simultaneously maximized both the sensitivity (77.8%) and specificity (80%) of the test was decreased by 71.1%. **D**. The area under the ROC curve of CA125 change values was 0.800 (95% CI: 0.537 to 1.063, *P* = 0.128); the optimal cutoff which simultaneously maximized both the sensitivity (100%) and specificity (60%) of the test was decreased by 40.0%.

To assess the ability of the changes of tumor markers to predict the pathologic complete and partial response to neoadjuvant chemotherapy, ROC curve was carried out (Figure 4). The area under the ROC curve of CA72-4 change values was 0.764 (95% CI: 0.584 to 0.945, *P* = 0.020); the optimal cutoff which simultaneously maximized both the sensitivity (77.8%) and specificity (80%) of the test was decreased by 71.1%. The area under the ROC curve of CA125 change values was 0.800 (95% CI: 0.537 to 1.063, *P* = 0.128); the optimal cutoff which simultaneously maximized both the sensitivity (100%) and specificity (60%) of the test was decreased by 40.0%.

To confirm the relationship between tumor marker decrease and pathologic response, Spearman correlation was performed;the correlation coefficient between CA72-4 decreased and pathologic response was 0.404, and the *P* value was 0.018. The correlation coefficient between CA125 decreased and pathologic response was 0.439, and the *P* value was 0.133.

### Association of pretreatment CEA levels with response to neoadjuvant chemotherapy

According to Table 1, the positivity rate of pretreatment CEA was higher when clinical disease progression and no pathologic response were present (*P* = 0.012 and *P* = 0.145). Figure 3 shows that serum levels of pretreatment CEA were higher in patients with clinical disease progression after neoadjuvant chemotherapy.

**Figure 5:**
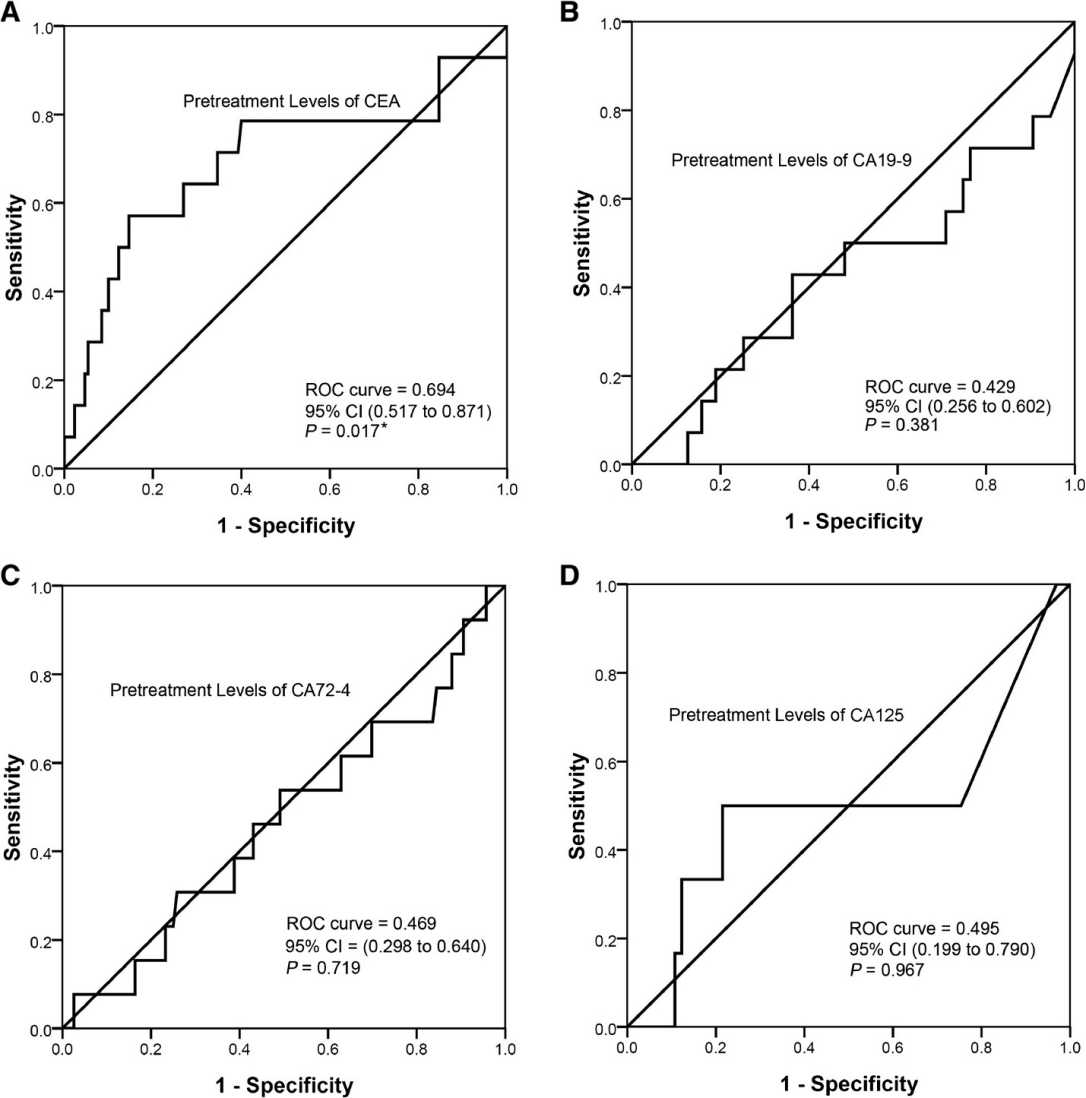
**ROC curves for pretreatment levels of tumor markers to predict the disease progression after neoadjuvant chemotherapy. A**. The area under the ROC curve of CEA pretreatment levels was 0.694 (95% CI: 0.517 to 0.871, *P* = 0.017); the cutoff that maximized both sensitivity (57.1%) and specificity (85.4%) of this test was 50 ng/ml. The correlation coefficient between CEA pretreatment levels and pathologic response was 0.199, and the *P* value was 0.017. **B**.**C**.**D** show the pretreatment levels of CA72-4, CA199, and CA125 were not statistically significant (*P* values were 0.381, 0.719, and 0.967 respectively).

To evaluate whether pretreatment levels of tumor markers can be used as marker of clinical disease progression after neoadjuvant chemotherapy, an ROC curve was carried out (Figure 5). The area under the ROC curve of CEA pretreatment levels was 0.694 (95% CI: 0.517 to 0.871, *P* = 0.017); the cutoff that maximized both sensitivity (57.1%) and specificity (85.4%) of this test was 50 ng/ml.

The correlation between pretreatment levels of tumor markers and pathologic response was also analyzed in our study. The correlation coefficient between CEA pretreatment levels and pathologic response was 0.199, and the *P* value was 0.017.

## Discussion

Gastric cancer is still a major health problem worldwide due to its frequency, poor prognosis, and limited treatment options. Despite efforts for early detection, most patients with gastric cancer continue to present with an advanced of the disease. To improve survival in these patients, preoperative neoadjuvant chemotherapy became an available option, and several studies have certified that neoadjuvant chemotherapy could down-stage patients, improve curative respectability, and improve survival rates [3, 5, 6, 17]. Despite these advantages, some patients still have with no response to neoadjuvant chemotherapy, to improve the quality of life of non-responders, reduce time until surgery, and reduce costs, the markers which can monitor the response to neoadjuvant chemotherapy are needed. Our study focuses on clinical utility of tumor markers CEA, CA19-9, CA72-4, and CA125 in gastric cancer patients with neoadjuvant chemotherapy, and the aim of the study is to measure whether these tumor markers might be useful in monitoring response and in predicting the prognosis of patients.

It is well known that the serum levels of various tumor markers such as CEA, CA19-9, CA72-4, and CA125 are elevated in patients with gastric cancer; however, there are still controversies in the clinical use of these tumor markers. The pretreatment positivity rates of tumor markers are different in previous studies., CEA positivity is normally reported at between 15.8 and 57.6%, CA19-9 at between 23.1 and 50%, and CA72-4 at between 18.6 and 58% [14, 18–22], and results of our study on the percentage of marker positivity are within these ranges (CEA: 37.7%; CA19-9: 23.1%; CA72-4: 42.1%; and CA125: 23.2%). As previously reported [19, 20, 22, 23], we also demonstrated that the combined evaluation of the markers in the serum showed a significant increase in the diagnostic sensitivity documented by the percentage of cases where the level of at least one marker was higher than its cut-off value.

To find out the correlation between pretreatment status of tumor markers and different clinicopathologic parameters, we performed a chi-square test, and found that CEA positivity was associated with lymph node involvement; our result was in line with a previous study [22]. In the literature, CA72-4 appeared to be the most sensitive and specific marker in gastric cancer patients, and its positivity was associated with advanced tumor stage, lymph node metastasis, and distant metastasis [18, 19, 21, 24–27]. Our results confirm that it was associated with advanced tumor stage and vascular invasion. In our study, we also found that the positivity of combined detection of four tumor markers was related to deeper tumor invasion, lymph node metastasis, and advance tumor stage. Through these results, we confirmed that the pretreatment levels of tumor markers such as CEA and CA72-4 can predict the biological behavior of gastric cancer, to some extent.

According to univariate and multivariate analyses, we found that patients with pretreatment positive values for tumor markers showed worse prognosis. Survival curves (Kaplan-Meier curves) according to pretreatment levels of CA19-9 and CA72-4 showed significant differences between positive and negative patients, and the two markers were also confirmed to be independent prognostic factors in multivariate analysis; our experience was consistent with previous reports [18–22, 28, 29]. Cox proportional hazards regression analysis indicated that patients with elevated levels of CA19-9 and CA72-4 had a 2.183-fold and 2.500-fold higher risk of death than patients with low levels, respectively. The reason preoperative levels of tumor markers influence long-term survival is not clear; a number of biological factors are probably involved [18, 21, 22].

Previous studies have confirmed that tumor markers can be predictors of the response to chemotherapy in patients with pancreatic cancer, breast cancer, epithelial ovarian cancer, and colorectal cancer [30, 34]; however, clinical studies evaluating the roles of tumor markers in the monitoring of chemotherapeutic efficacy in patients with gastric cancer are limited [9]. In the present study, we pay attention to the utility of tumor markers in monitoring the response to neoadjuvant chemotherapy in patients with gastric cancer. We found that mean levels of tumor markers were decreased after neoadjuvant chemotherapy, especially in the disease control group, which included patients with complete response (CR), partial response (PR), and stable disease (SD); mean levels decreased more significantly than disease progression group. The decreases in CEA, CA72-4, and CA125 levels achieved statistical significance; we saw the same tendency in CA19-9 levels but the difference did not achieve statistical significance. To explain this phenomenon, we reviewed the medical records and found that pretreatment CA19-9 levels in a small number of patients were extremely elevated; all these patients appeared to be in the disease control after neoadjuvant chemotherapy group, and this may be the reason why the decrease of CA19-9 levels did not achieve statistical significance.

Based on the result that tumor markers decreased more significantly in patients with disease control, we did an ROC curve to assess the ability of changes in tumor markers to predict response to neoadjuvant chemotherapy. We found that the decrease of CA72-4 after neoadjuvant chemotherapy could predict pathologic response, and the cutoff value decreased by 71.1%. This means that if CA72-4 levels decrease by more than 71.1%, the patient has more chance to achieve pCR and pPR. We also found that the area under the ROC curve of CA24-2 change values was 0.800; the cutoff value was decreased 40.0% but the difference did not achieve statistical significance. Our inability to demonstrate that the decrease of CA125 can also predict pathologic response to neoadjuvant chemotherapy may be due to the relatively small number of patients analyzed.

Using chi-square analysis, we found positive pretreatment levels of CEA were associated with clinical disease progression after neoadjuvant chemotherapy; and using t-test, we found CEA levels in the disease progression group were higher than the disease control group. Therefore, we speculated that high pretreatment level of CEA may predict clinical disease progression. In order to confirm this, we carried out an ROC curve and found pretreatment level of CEA can predict clinical disease progression after neoadjuvant chemotherapy; the cutoff value was 50 ng/ml. Therefore, if the pretreatment level of a patient is more than 50 ng/ml, he has a higher of being a non-responder.

It is well known that elevated marker levels decrease after curative resection of the tumor, only to elevate again on recurrence [9, 35, 36]. Therefore some researchers have postulated that the change in these tumor markers would reflect the relative tumor burden in individual patients after chemotherapy, when the marker levels had been elevated prior to chemotherapy [9]. We are agree with this speculation as tumor markers can be produced directly by the tumor or non-tumor cells as a response to the presence of a tumor, so a high level of tumor marker may mean a large tumor burden. This may be the reason why high pretreatment levels of tumor markers can predict poor prognosis, and why high CEA levels can predict poor response to neoadjuvant chemotherapy. Tumor burden will be reduced by effective treatment such as neoadjuvant chemotherapy, and tumor shrinkage always accompanies a drop in marker levels, therefore we can see a decrease in tumor markers after neoadjuvant chemotherapy, especially in patients with disease control.

The limitations of our study include that the number of patients analyzed here is very small and missing values still exist. Due to the low sensitivity and specificity, these tumor markers cannot replace other detection methods, such as pathological examination and imaging studies.

## Conclusions

In conclusion, the measurement of tumor markers might be useful in the monitoring of response, and in the prediction of prognosis in patients treated with neoadjuvant chemotherapy. This is especially true in the cases in which disease is difficult to evaluate by imaging studies. Further studies are required to confirm these findings.

## Abbreviations

5-FU: 5-Flourouracil
AJCC: American Joint Committee on Cancer
AUC: Area Under the Curve
CA19-9: Carbohydrate Antigen 19–9
CA242: Carbohydrate Antigen 242
CA72-4: Carbohydrate Antigen 72–4
CEA: Carcinoembryonic Antigen
CR: Complete Response
CT: Computed Tomography
CTCs: Circulating Tumor Cells
DC: Disease Control
EUS: Endoscopic Ultrasonography
LV: Leucovorin
MDT: Multidisciplinary Team
NAC: Neoadjuvant Chemotherapy
NCCN: National Comprehensive Cancer Network
NR: No Response
OXA: Oxaliplatin
PD: Progressive Disease
PR: Partial Response
ROC Curve: Receiver Operating Characteristic Curve
SD: Stable Disease
UICC: International Union Against Cancer
WHO: World Health Organization.
